# Pain Decrement Using Radiofrequency Therapy After Knee Platelet-Rich Plasma Injections Within First 72 h in Active Populations with Patellar Chondropathy

**DOI:** 10.3390/jcm14020544

**Published:** 2025-01-16

**Authors:** Ferran Abat, Jordi Torras, Alba Garcia, Enrique Jordán, Matías Roby, Roberto Yáñez, Carlos De la Fuente

**Affiliations:** 1 GRACIS Research Group (GRC 01604), Sports Orthopaedic Department, ReSport Clinic, Higher School of Health Sciences Tecnocampus, Pompeu Fabra University, 08029 Barcelona, Spain; 2 Physiotherapy Department, ReSport Clinic, Blanquerna School of Health Science, Universitat Ramon Llull, 08022 Barcelona, Spain; torras@resportclinic.com (J.T.); garcia@resportclinic.com (A.G.); jordan@resportclinic.com (E.J.); 3 Innovation Center, Clínica MEDS, Santiago 7550615, Chile; matias.roby@meds.cl; 4 Knee Orthopedics Service, Clínica MEDS, Santiago 7550615, Chile; roberto.yanez@meds.cl; 5 Exercise and Rehabilitation Sciences Institute, Postgraduate, Faculty of Rehabilitation Sciences, Universidad Andres Bello, Santiago 7591538, Chile

**Keywords:** physical agents, electrotherapy, high frequency, knee, patella, anterior knee pain

## Abstract

**Objective:** To determine whether 448 kHz capacitive-resistive monopolar radiofrequency (CRMR) after platelet-rich-plasma (PRP) injections can further reduce pain sensation within the first 72 h in an active population with patellar chondropathy. **Methods:** One-hundred fifty-three active patients with patellar chondropathy grade II-III were followed for three days after PRP injections with and without CRMR under a control–placebo study. They were clinically evaluated for pain sensation using a visual analog scale ranging from zero (no pain sensation) to ten (highest pain sensation). Pain sensation was described using medians and analyzed through the Friedman and Conover test for within-group comparison (pre-intervention, and 24, 48, and 72 h post-intervention) and the Mann–Whitney test for between-group comparisons (Intervention vs. Placebo) with α = 5% and 1−β = 80%. **Results:** The placebo group showed statistical significance between pre-intervention and 24 h (Δ = −2.0 pts, *p* < 0.001), baseline and 48 h (Δ = −2.0 pts, *p* < 0.001), baseline and 72 h (Δ = −3.0 pts, *p* < 0.001), 24 h and 48 h (Δ = 0.0 pts, *p* < 0.016), and 24 h and 72 h (Δ = −1.0 pts, *p* < 0.001). The radiofrequency group showed statistical significance between baseline and 24 h (Δ = −7.0 pts, *p* < 0.001), baseline and 48 h (Δ = −7.0 pts, *p* < 0.001), baseline and 72 h (Δ = −8.0 pts, *p* < 0.001), 24 h and 72 h (Δ = −1.0 pts, *p* < 0.001), and 48 h and 72 h (Δ = −1.0 pts, *p* < 0.001). The placebo and radiofrequency groups were significantly different at 24 h (Δ = 4.0 pts, *p* < 0.001), 48 h (Δ = 4.0 pts, *p* < 0.001), and 72 h (Δ = 4.0 pts, *p* < 0.001). **Conclusions:** CRMR therapy administered after knee intra-articular injections of PRP within the first 72 h in active populations with patellar chondropathy reduces pain sensation with a median difference of 8.0 pts compared to baseline and 4.0 pts compared to placebo group.

## 1. Introduction

Patellofemoral chondropathy results from the degeneration or loss of articular cartilage in the patella, with or without involvement of the trochlear groove [1,2]. It is more common than tibiofemoral osteoarthritis and particularly affects females over the age of 50 [1,2] as well as active populations [3]. Unfortunately, patellofemoral chondropathy is associated with functional impairments and can also be linked to patellofemoral pain syndrome, which may begin in younger years [4].

Concentrated intra-articular platelet-rich plasma (PRP) therapy is a regenerative approach that may enhance healing and promote cartilage regeneration [5]. After centrifugation, PRP is rich in growth factors such as transforming growth factor-beta, insulin-like growth factor, platelet-derived growth factor, vascular endothelial growth factor, and epidermal growth factor. PRP therapy has demonstrated clinical benefits for knee chondropathy, including pain relief and improved knee function [5,6,7]. It plays a crucial role in regulating chondral homeostasis and supporting both healing and chondrogenesis [7].

Recent findings indicate that an intra-articular injection of PRP produces outcomes comparable to those of a single corticosteroid injection in late-stage knee osteoarthritis [6]. Consequently, exploring complementary non-invasive treatments for pain relief is crucial to managing acute and persistent pain effectively, supporting functional recovery, and preventing overmedication. Since 448 kHz capacitive-resistive monopolar radiofrequency (CRMR) has demonstrated benefits in enhancing blood circulation, modulating nociceptive pain, and tissue healing, it has shown promise in relieving chronic pain and improving functionality in conditions such as knee osteoarthritis [8], chronic pain pelvis syndrome, and low back pain [9]. Thus, the combination PRP injection with CRMR may reduce acute pain sensation in patients with patellar chondropathy and performed within the first 72 h is crucial to avoid arthrogenic muscle inhibition [10,11], starting an early muscle re-education as far as possible [12] with overmedication prevention. Furthermore, the literature has established a clinically important change as ≥30 mm or 3 pts) [13].

Consequently, we aim to determine whether CRMR after PRP injections can further reduce pain sensation within the first 72 h in an active population with patellar chondropathy grade II–III.

## 2. Materials and Methods

### 2.1. Study Design

**Figure 1:**
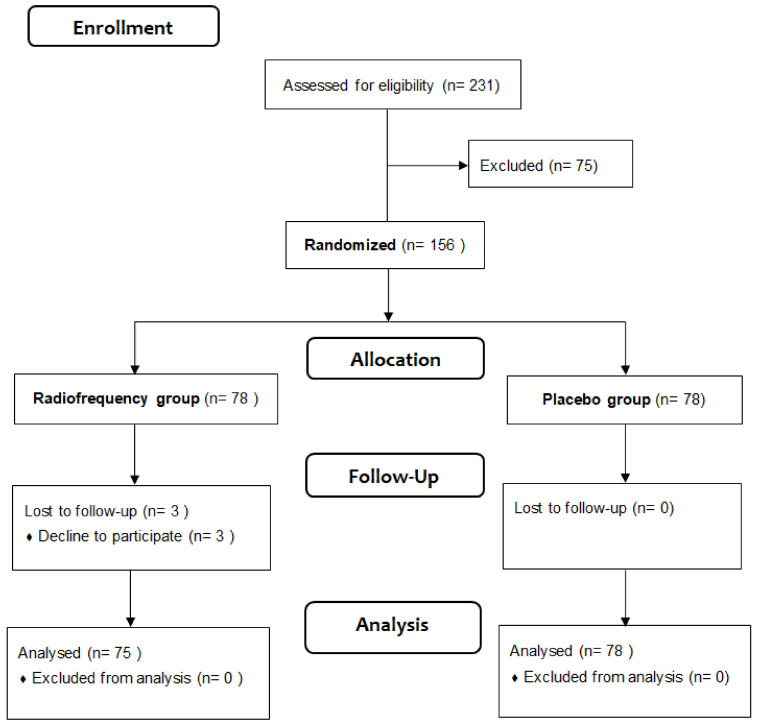
Enrollment, follow-up, and analysis of patients based on CONSORT methodological guideline.

In this prospective control–placebo study, patients with patellar chondropathy grade II–III were followed up for three days after PRP injections with and without radiofrequency treatment immediately applied (5 min after PRP). Patients were randomized, as Figure 1 shows. Pain sensation was clinically evaluated using a visual analog scale from zero (no pain sensation) to ten (highest pain sensation) before the intervention (baseline), 24 h, 48 h, and 72 h post-interventions.

### 2.2. Participants

From August 2019 to December 2023, one-hundred fifty-three adult patients (aged 43.8 ± 12.5 years) were included in the study based on eligibility study criteria (Figure 1). These inclusion criteria were: (i) age between 18 and 70 years, (ii) patellar chondropathy grade II–III diagnosed by magnetic resonance according to the American College of Rheumatology [14], (iii) active population (engaging in a minimum of 30 min of planned physical activity per week), (iv) functional limitation due to patellar tendon pain during knee flexion, and (v) treatment in our center. The exclusion criteria included radiotherapy contraindications such as (i) pregnancy, (iii) active cancer or infection, (iii) pacemaker or electronics implants, (iv) metal implants in the affected knee, (v) neurological deterioration, (vi) active skin lesions in the knee region, (vii) heat hypersensitivity, and (viii) any surgical procedure or pathological condition in the affected region of intervention.

The analyzed sample consisted of an active population, including 91 men and 60 women, who participated in padel tennis (*n* = 25), gym activities (*n* = 24), cycling (*n* = 19), running (*n* = 14), triathlon (*n* = 13), walking (*n* = 10), basketball (*n* = 10), dancing (*n* = 8), rugby (*n* = 4), and other sports (*n* = 26).

This study was approved by the institutional review board (CER-FCSB n.20191202) and conducted in accordance with the Helsinki Declaration. All the patients provided written consent to participate in the study.

### 2.3. Sample Size

The sample size was estimated in 156 patients, accounting for an effect size of 0.1 (small η^2^ equal 0.01), an alpha of 5%, a statistical power of 80%, a 13% attrition rate, two groups, four-time points, and repeated measurements within-between interaction.

### 2.4. Randomization and Allocation

Participants were randomized in a 1:1 ratio to integrate the placebo (without intervention) or radiofrequency group (with intervention) before beginning the study (Figure 1).

### 2.5. Patellar Chondropathy Diagnosis

The clinical diagnosis was made by the same medical doctor (FA). The clinical signs and symptoms included persistent joint pain that typically worsens with activity and improves with rest, as well as morning or post-inactivity knee stiffness lasting less than 30 min. Additional indicators, such as crepitus, tenderness along the joint line, bony enlargement, and restricted range of motion, reflected degenerative joint processes and confirmed the clinical diagnosis.

The diagnosis was further validated using radiographic and magnetic resonance images (MRI). Radiographic findings included diminished joint space, osteophyte formation, subchondral sclerosis, and subchondral cysts, indicative of cartilage loss and bone remodeling in response to joint stress. MRI findings included cartilage thinning or defects, subchondral bone marrow lesions, osteophytes, joint effusion, and synovitis.

### 2.6. PRP Intervention

The clinical protocol used for PRP injection preparation involved drawing around 20 mL of venous blood from the median cephalic vein, aiming to obtain a final injectable volume between 3 mL of concentrated platelets (300,000 platelets/µL, reaching a concentration factor of 2.6) and growth factors (leukocyte-poor). The venous blood was processed using a centrifugation technique (PRGF–ENDORET^®^ Technology, BTI Biotechnology Institute, Gasteiz, Spain). Centrifugation was configured for 8 min at 580× *g* to separate blood cells from plasma through tangential forces. Leukocytes were marked 0.2 to 0.3 mL above the red blood cells for extraction. This initial procedure, from blood collection to leukocyte fractioning, was completed within 30 min. After the fractioning, the remaining process was completed in less than 5 min. The plasma was then activated using 20 μL of 10% calcium chloride per millimeter of plasma immediately before intra-articular infiltration [15].

Once the PRP was prepared for injection during an in-hospital medical consultation by the same medical doctor (FA), each patient was positioned in a supine position with the knee slightly flexed to open the frontal knee joint space, minimizing the risk of cartilage damage at the injection is delivered. Ice was applied to the knee ten minutes before the injection.

The knee region was sterilized with an antiseptic solution, and sterile drapes were placed around the knee to maintain an aseptic field. Then, a high-definition musculoskeletal ultrasound (Canon Medical Systems, Corp., Tochigi, Japan) operating at 14 MHz with a 58 mm linear probe and Doppler color mode was used for continuous needle path visualization. The injection was administrated on the superolateral side at the joint line beneath the proximal patellar tendon using a 21-gauge sterile needle inserted at a 30–45-degree angle.

The PRP injection was distributed within the joint, targeting the damaged patellar surface under ultrasound guidance. The PRP is entered gradually within the joint, and later, with joint mobilization, it is distributed throughout the joint. Finally, ultrasound imaging was used to confirm PRP distribution within the joint before needle removal [16].

### 2.7. Radiofrequency Intervention and Placebo

The CRMR intervention followed a previously described procedure [17] and was carried out after 5 min of PRP intervention. A session of 15 min, using high frequency current (448 kHz) CRMR (Indiba activ CT8, INDIBA, Sant Quirze del Vallès, Spain) at 200 W. It included 5 min of capacitive mode, which primarily targets more superficial and better vascularized anatomical areas, and 10 min of resistive mode, where the current flows through tissues with higher impedance, often associated with reduced hydration-vascularization. This intervention was performed after knee PRP infiltration.

During the session, the patient lay in a supine position with the knee fully extended. Two electrodes were placed to create a current circuitry encompassing the knee. The active electrode was applied directly to the knee, and the return electrode was placed on the posterior side of the lower limb, between the ankle and knee. Both electrodes maintained full contact with the skin under comfortable pressure to ensure even distribution of the electrical current and to prevent localized electric concentration (skin burning).

For the placebo treatment, the setup mirrored the CRMR procedure, but the equipment was turned off. Patients were blinded as to the kind of treatment mode they received (capacitive, resistive, or placebo).

### 2.8. Clinical Pain Assessment

Pain sensation was evaluated using a visual analog scale [18]. The scale ranged between zero pts (no pain sensation) to ten pts (highest pain sensation) with a resolution of 1 point. The same evaluator provided consistent instructions at the start of each pain assessment and collected the scores for each patient immediately following the PRP and CRMR intervention at 24, 48, and 72 h.

### 2.9. Data Analysis

Pain sensation was summarized with median and interquartile range after confirming a non-normal distribution of data through the Shapiro–Wilk test and testing for heteroscedasticity with Levene’s test. Data were analyzed through a Friedman and Conover test for within-group comparisons (pre-intervention (baseline), and 24, 48, and 72 h post-intervention), while the Mann–Whitney test was applied for between-group comparisons (radiofrequency vs. Placebo group). All tests were conducted with a statistical α level of 5% and statistical power (1−β) of 80%. All statistical estimations were performed using the open-source software JASP 0.18.3 (University of Amsterdam, Amsterdam, The Netherlands).

## 3. Results

**Figure 2:**
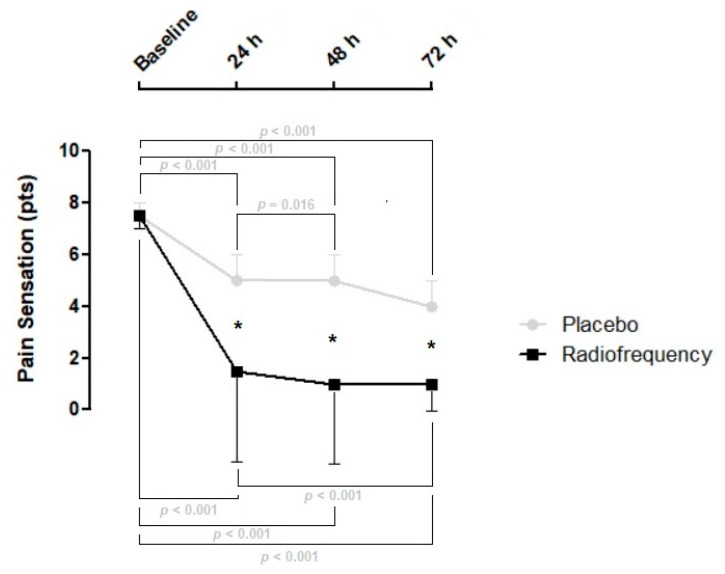
Multiple comparisons of pain sensation outcomes. The figure shows the placebo group in a gray line, while the radiofrequency group is shown in a black line. Within and between effects can be visualized. Data are summarized as median (circle and square symbols) and inter-quartile range (half of the error bar). Statistical significance between groups is expressed with an asterisk representing a *p* < 0.001.

Descriptive statistics of the study are summarized in Table 1. There is a time effect for the radiofrequency group (χ^2^: 190.9, W: 0.82, *p* < 0.001) and the placebo group (χ^2^: 171.9, W: 0.76, *p* < 0.001), see Figure 2.

## 4. Discussion

The main findings of our manuscript were that CRMR therapy after knee intra-articular knee injections of PRP within the first 72 h in an active population with patellar chondropathy grade II–III reduces pain sensation with a median difference of 8.0 pts compared to baseline and 4.0 pts compared to placebo dose. These reductions in pain sensation represent clinically important changes (≥30 mm [13]), helping to prevent prolonged overuse of pain relief medications and facilitating early patellofemoral functional rehabilitation without persistent pain. Managing pain from patellar chondropathy typically involves a multifaceted approach, including physical therapy, rest, activity modification, and, in some cases, anti-inflammatory medications. Strengthening exercises targeting the quadriceps and improving flexibility are crucial for enhancing patellar tracking and alleviating discomfort. As such, our innovative pain reduction strategy is clinically significant for pain management.

The most significant change in pain sensation occurred within the first 24 h in both groups, following a similar pattern of pain reduction over 72 h period after PRP injections. However, the radiofrequency group consistently showed a greater reduction in pain sensation compared to the placebo group at all time points in our study. These findings align with the pain-relief effects reported for PRP knee injections when compared to placebo interventions [19]. Meta-analytic evidence has demonstrated similar improvements starting 6 weeks post PRP injections for both single and double PRP spin techniques [19]. The observed acute pain reduction in both groups during the first 72 h may be attributed to PRP’s effect on blocking serotonin reuptake, allowing it to act as an acute analgesic, diminishing nerve sensitivity [20].

Evidence from pulsed radiofrequency studies suggests an enhancement of the top-down noradrenergic and serotonergic inhibitory pathways involved in the modulation of pain [21,22,23]. In our study, the interaction between PRP and CRMR therapy likely contributed to the greater reduction in pain sensation observed within 72 h. Our finding aligns with previous studies that found an acute decrease in pain sensation of ~66 to 68% [8,24].

The reduced pain sensation is crucial for initiating early motor re-education therapy and therapeutic exercises, enabling patients to enhance functionality and prevent arthrogenic muscular inhibition [8,10,11,25]. Additionally, this reduction in pain can support early return to work and reduce dependency on pain relief medications. Overuse of drugs like non-steroidal anti-inflammatory drugs (NSAIDs) can lead to complications, such as acute liver injury [26]. Thus, our pain sensation decreases within the first 72 h aligns with past patient-reported feedback, indicating reduced reliance on pain medication [8].

Potential mechanisms underlying our results include improvements in pain transmission [21] rather than structural tissue changes, such as matrix synthesis, as our study duration was limited to 72 h. Local effects such as pH normalization, increased nutrients and oxygen delivery, and improved microenvironment conditions may have contributed [24]. However, future controlled causal experiments are needed to clarify the causal mechanisms behind the acute effects found in this study. Also, research design offering a PRP group alone can be beneficial in separating PRP, RF, and PRP+RF effects.

This study is not without limitations. More time for the follow-up would be useful to confirm the stability of pain sensation decreases across the rehabilitation. Also, blood samples and images should be important in exploring systemic and structural adaptations.

## 5. Conclusions

CRMR therapy after knee intra-articular injections of PRP within the first 72 h in active populations with patellar chondropathy grade II–III decreases pain sensation with a median difference of 8.0 pts and 4.0 pts compared to baseline and placebo. This acute decrease in pain sensation is a clinically important change (≥30 mm or 3 pts), which can impact the prevention of prolonged overuse of pain relief medications and promote the beginning of patellofemoral functional rehabilitation without the presence of persistent pain.

## Figures and Tables

**Table 1:** Descriptive statistics of pain sensation outcomes measure in pts.

	Placebo					Radiofrequency					Radiofrequency—Placebo			
	Median [IQR]	Within-Group Δ				Median [IQR]	Within-Group Δ				Between-Group Δ			
		**Baseline**	**24 h**	**48 h**	**72 h**		**Baseline**	**24 h**	**48 h**	**72 h**	**Baseline**	**24 h**	**48 h**	**72 h**
**Baseline**	7.0 [1.8]	-	−2.0	−2.0	−3.0	8.0 [1.0]	-	−7.0	−7.0	−8.0	1.0	−6.0	−6.0	−7.0
**24 h**	5.0 [2.0]		-	0.0	−1.0	1.0 [5.0]		-	0.0	−1.0	3.0	−4.0	−4.0	−5.0
**48 h**	5.0 [3.0]			-	−1.0	1.0 [3.5]			-	−1.0	3.0	−4.0	−4.0	−5.0
**72 h**	4.0 [3.0]				-	0.0 [2.0]				-	4.0	−3.0	−3.0	−4.0

Δ: Delta (difference), IQR: Interquartile range, ●: clinically important difference (≥|3| pts), and ●: No clinically important difference (<|3| pts). The Δ values of the symmetrical square matrix of within-group are omitted.

## Data Availability

The analyzed datasets during the current study are available from the corresponding author F.A. upon request.
